# Apo-9′-Fucoxanthinone, Isolated from *Sargassum muticum*, Inhibits CpG-Induced Inflammatory Response by Attenuating the Mitogen-Activated Protein Kinase Pathway

**DOI:** 10.3390/md11093272

**Published:** 2013-08-27

**Authors:** Doobyeong Chae, Zahid Manzoor, Sung Chun Kim, Sohyun Kim, Tae-Heon Oh, Eun-Sook Yoo, Hee-Kyoung Kang, Jin-Won Hyun, Nam Ho Lee, Mi-Hee Ko, Young-Sang Koh

**Affiliations:** 1 School of Medicine, Jeju National University, Jeju 690-756, Korea; E-Mails: enqud2@gmail.com (D.C.); whitebrands57@yahoo.com (Z.M.); just_so@naver.com (S.K.); eunsyoo@jejunu.ac.kr (E.-S.Y.); pharmkhk@jejunu.ac.kr (H.-K.K.); jinwonh@jejunu.ac.kr (J.-W.H.); 2 Institute of Medical Science, Jeju National University, Jeju 690-756, Korea; 3 Department of Chemistry, College of Natural Sciences, Jeju National University, Jeju 690-756, Korea; E-Mails: ksctotoro@naver.com (S.C.K.); island75@jejunu.ac.kr (T.-H.O.); namho@jejunu.ac.kr (N.H.L.); 4 Jeju Biodiversity Research Institute, Jeju Technopark, Jeju 699-943, Korea; E-Mail: miheeko@jejutp.or.kr

**Keywords:** *Sargassum muticum*, apo-9′-fucoxanthinone, activator protein-1, pro-inflammatory cytokine, ERK1/2, inflammation

## Abstract

*Sargassum muticum* (*S. muticum*) is a brown edible alga and widely distributed in Korea. This report was designed to evaluate the anti-inflammatory properties of apo-9′-fucoxanthinone (APO-9′) isolated from *S. muticum* on pro-inflammatory cytokine production*.* *S. muticum* extract (SME) exhibited significant inhibitory effects on pro-inflammatory cytokine production in bone marrow-derived macrophages (BMDMs) and dendritic cells (BMDCs). APO-9′ pre-treatment in the CpG DNA-stimulated BMDMs and BMDCs showed a strong dose-dependent inhibitory effect on interleukin (IL)-12 p40, IL-6 and tumor necrosis factor (TNF)-α production with IC_50_ values ranging from 5.31 to 13.79. It exhibited a strong inhibitory effect on the phosphorylation of ERK1/2 and on activator protein (AP)-1 reporter activity. APO-9′ pre-treatment exhibited significant inhibition of CpG DNA-induced production of inducible nitric oxide synthase. Taken together, these data suggest that SME and APO-9′ have a significant anti-inflammatory property and warrant further studies concerning the potentials of SME and APO-9′ for medicinal use.

## 1. Introduction

Toll-like receptors (TLRs) play a critical role in innate host immune response against pathogens through recognition of molecular patterns exclusively present in microorganisms [1]. The TLR family members can recognize different classes of pathogens and coordinate suitable innate and adaptive immune responses [2]. Bone marrow-derived macrophages (BMDMs) and dendritic cells (BMDCs) are vital cellular components of the innate immune system and play an important role in combating different pathogens [3]. Stimulation of macrophage and dendritic cells through TLRs result in production of interleukin (IL)-12 p40, IL-6 and tumor necrosis factor (TNF)-α [1,4]. Detection of pathogen-associated molecular patterns (PAMPs) by TLRs triggers activation of downstream signaling cascades, including nuclear factor kappa-light-chain-enhancer of activated B-cells (NF-κB) and the mitogen-activated protein kinase (MAPK) pathway, leading to production of pro-inflammatory cytokines [5].

MAPK is one of the main signal transduction pathways that belong to a large family of serine/threonine kinases. MAPKs have three well-characterized subfamilies, including extracellular signal-regulated kinases (ERK), the c-Jun *N*-terminal kinases (JNK) and the p38 family of kinases (p38 MAPKs) [6,7]. Activation of ERK1/2 leads to phosphorylation of different substrates, which includes some membrane proteins and activator protein-1 [8]. ERKs are involved in expression of multiple cytokines, which, in turn, play an important role in innate immunity [9].

Microbial DNA sequences containing unmethylated CpG dinucleotides activate Toll-like receptor 9 expressed in BMDMs and BMDCs [10,11]. However, the TLR9-mediated immune cells’ activation is found to be triggered by mammalian self-DNA incorporated into immune complexes in some autoimmune diseases [10,12].

Inducible nitric oxide synthase (iNOS) is a family of enzymes that catalyzes the production of nitric oxide from l-arginine, which is an important cellular signaling molecule [13,14]. Activated iNOS plays an important role in inflammatory and autoimmune diseases [15,16]. Therefore, the production of inflammatory mediators is an important target in the treatment of inflammatory diseases.

Recently, many marine resources have attracted attention in the search for bioactive compounds of high nutritional value and pharmaceutical potential [17]. *Sargassum muticum* (*S. muticum*) is a brown edible alga and widely distributed on the seashores of Korea. A previous study revealed that *S. muticum* extract (SME) has various biological activities, including antioxidant, antimicrobial and anti-inflammatory properties [18,19]. As a part of our continuing research to evaluate the biological activities, we have screened the anti-inflammatory effects of marine algae collected off the Jeju coastal area and found that *S. muticum* has potent anti-inflammatory activity. Fucoxanthin is one of the major carotenoids, known for its antioxidant, anti-inflammatory, anti-obesity, antitumor and UV-preventative activities [20,21,22,23]. It has been reported that marine *Dinoflagellate amphidinium* contains apo-9′-fucoxanthinone (APO-9′) [24]. APO-9′ was previously reported as a degradative product of fucoxanthin [25]. However, the effect of APO-9′ on innate immune response has been barely studied in terms of its influence on primary murine BMDMs and BMDCs. Therefore, in this study, we report the isolation and evaluation of the anti-inflammatory effects of APO-9′ from *S. muticum*.

## 2. Results

### 2.1. Inhibitory Effects of SME on Pro-Inflammatory Cytokine Production in CpG DNA-Stimulated BMDMs and BMDCs

**Figure 1:**
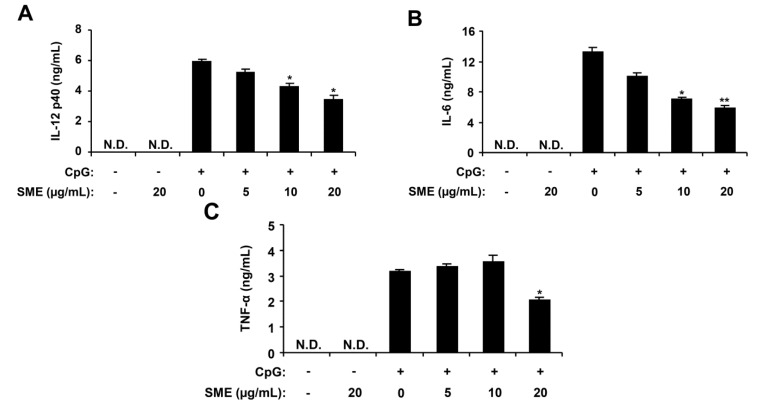
Inhibitory effects of *Sargassum muticum* extract (SME) on pro-inflammatory cytokine production in CpG DNA-stimulated bone marrow-derived macrophages (BMDMs). BMDMs were treated with SME at the indicated doses for 1 h before stimulation with CpG DNA (1 μM). The concentrations of murine IL-12 p40 (**A**), IL-6 (**B**), and TNF-α (**C**) in the culture supernatants were determined by enzyme-linked immunosorbent assay (ELISA). Data are representative of three independent experiments. N.D., not detectable; SME, *S. muticum* extract. * *p* < 0.05, ** *p* < 0.01 *vs*. SME-untreated cells in the presence of CpG DNA.

**Figure 2:**
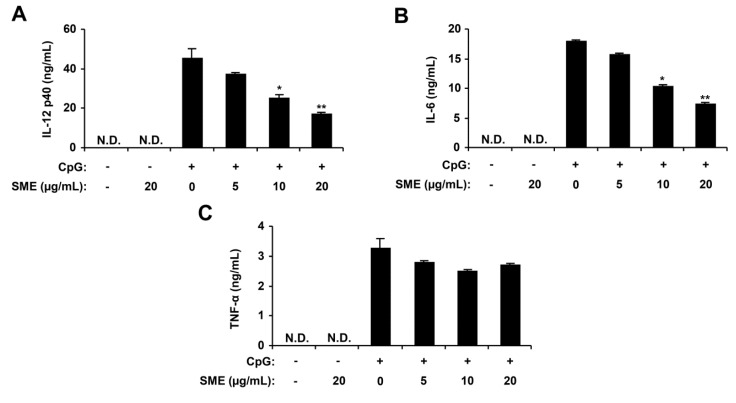
Inhibitory effects of SME on pro-inflammatory cytokine production in CpG DNA-stimulated bone marrow-derived dendritic cells (BMDCs). BMDCs were treated with SME at the indicated doses for 1 h before stimulation with CpG DNA (1 μM). The concentrations of murine IL-12 p40 (**A**), IL-6 (**B**) and TNF-α (**C**) in the culture supernatants were determined by ELISA. Data are representative of three independent experiments. N.D., not detectable; SME, *S. muticum* extract. * *p* < 0.05, ** *p* < 0.01 *vs*. SME-untreated cells in the presence of CpG DNA.

Macrophages and dendritic cells have a major role in the production of key cytokines, including IL-12 p40, IL-6 and TNF-α [1,26]. To assess the extract for anti-inflammatory activity, the *S. muticum* extract (SME) was tested for the inhibitory effects on CpG DNA-stimulated IL-12 p40, IL-6 and TNF-α production in BMDMs and BMDCs. To confirm the anti-inflammatory activity of SME, cell viability was simultaneously determined by using colorimetric 3-(4,5-dimethyl-2,5 thiazolyl)-2,5 diphenyl tetrazolium bromide (MTT) assay, and as a result, SME had no effect on the viability of BMDMs and BMDCs at the indicated concentrations (data not shown). SME treatment alone exhibited no production of cytokines (data not shown). CpG DNA induced a significant increase of IL-12 p40, IL-6 and TNF-α production in BMDMs and BMDCs. SME pre-treatment strongly inhibited IL-12 p40, IL-6 and TNF-α production in the CpG DNA-stimulated BMDMs with IC_50_ values of 27.18, 13.95 and 27.08 μg/mL, respectively (Figure 1). Similarly, it strongly inhibited IL-12 p40 and IL-6 production in CpG DNA-stimulated BMDCs with IC_50_ values of 13.0 and 14.27 μg/mL, respectively. However, it did not significantly inhibited TNF-α production (IC_50_ > 100 μg/mL, Figure 2). Taken together, these data indicate that SME had an inhibitory effect on cytokine production in CpG DNA-stimulated BMDMs and BMDCs.

### 2.2. Inhibitory Effects of APO-9′ on Pro-Inflammatory Cytokine Production in CpG DNA-Stimulated BMDMs and BMDCs

**Figure 3:**
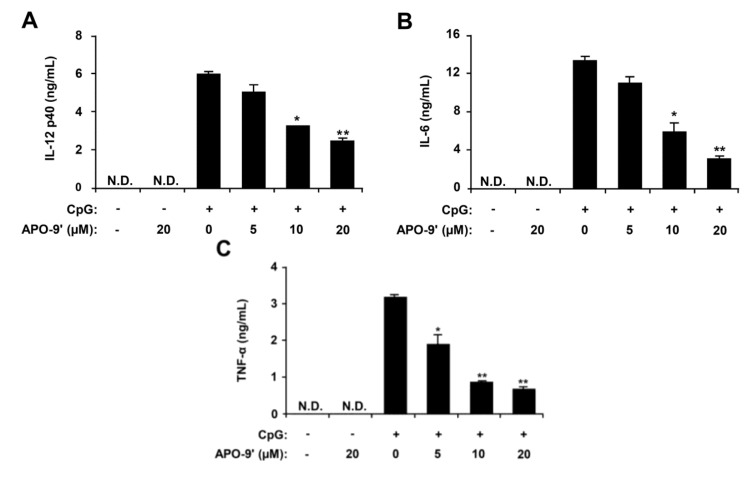
Inhibitory effects of apo-9′-fucoxanthinone (APO-9′) on pro-inflammatory cytokine production in CpG DNA-stimulated BMDMs. BMDMs were treated with APO-9′ at the indicated doses for 1 h before stimulation with CpG DNA (1 μM). The concentrations of murine IL-12 p40 (**A**), IL-6 (**B**) and TNF-α (**C**) in the culture supernatants were determined by ELISA. Data are representative of three independent experiments. APO-9′, Apo-9′-fucoxanthinone; N.D., not detectable. * *p* < 0.05, ** *p* < 0.01 *vs*. APO-9′-untreated cells in the presence of CpG DNA.

**Figure 4:**
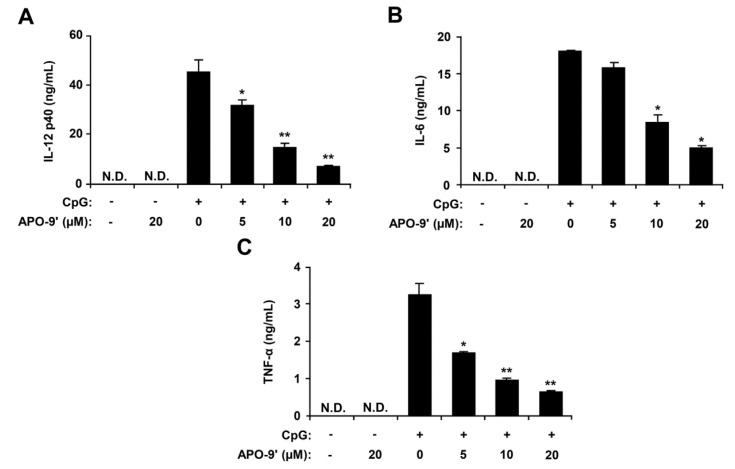
Inhibitory effects of APO-9′ on pro-inflammatory cytokine production in CpG DNA-stimulated BMDCs. BMDCs were treated with APO-9′ at the indicated doses for 1 h before stimulation with CpG DNA (1 μM). The concentrations of murine IL-12 p40 (**A**), IL-6 (**B**) and TNF-α (**C**) in the culture supernatants were determined by ELISA. Data are representative of three independent experiments. APO-9′, Apo-9′-fucoxanthinone; N.D., not detectable. * *p* < 0.05, ** *p* < 0.01 *vs*. APO-9′-untreated cells in the presence of CpG DNA.

To search the active components from *S. muticum*, SME was successively partitioned into *n*-hexane, methylene chloride, ethyl acetate, *n*-butanol and water fractions. *N*-hexane, ethyl acetate, *n*-butanol and water fractions have not much anti-inflammatory activity as compared to the methylene chloride fraction. Therefore, the methylene chloride fraction was further chromatographed using celite and normal phase silica gel. The identity of the purified compound was confirmed as APO-9′ by comparing its ^1^H- and ^13^C-NMR data with those reported in the literature [24]. To investigate the anti-inflammatory activity, APO-9′ was tested for inhibitory effects on CpG DNA-stimulated IL-12 p40, IL-6 and TNF-α production in BMDMs and BMDCs. APO-9′ treatment alone exhibited no production of pro-inflammatory cytokines in BMDMs and BMDCs (data not shown). CpG DNA-stimulation showed a substantial increase in the production of IL-12 p40, IL-6 and TNF-α in BMDMs and BMDCs. APO-9′ pre-treatment strongly inhibited IL-12 p40, IL-6 and TNF-α production in CpG DNA-stimulated BMDMs with IC_50_ values of 13.79, 8.4 and 6.17 μM, respectively (Figure 3). Similarly, it strongly inhibited IL-12 p40, IL-6 and TNF-α production in CpG DNA-stimulated BMDCs with IC_50_ values of 7.67, 8.92 and 5.31 μM, respectively (Figure 4). To confirm the anti-inflammatory activity of APO-9′, cell viability was simultaneously determined by using colorimetric MTT assay, and as a result, APO-9′ had no effect on the viability of BMDMs and BMDCs at the indicated concentrations (data not shown). These data indicate that APO-9′ had an inhibitory effect on pro-inflammatory cytokine production in CpG DNA-stimulated BMDMs and BMDCs.

### 2.3.Effects of APO-9′ on the Phosphorylation of MAPK and the Degradation of IκBα by CpG-Stimulated BMDMs

**Figure 5:**
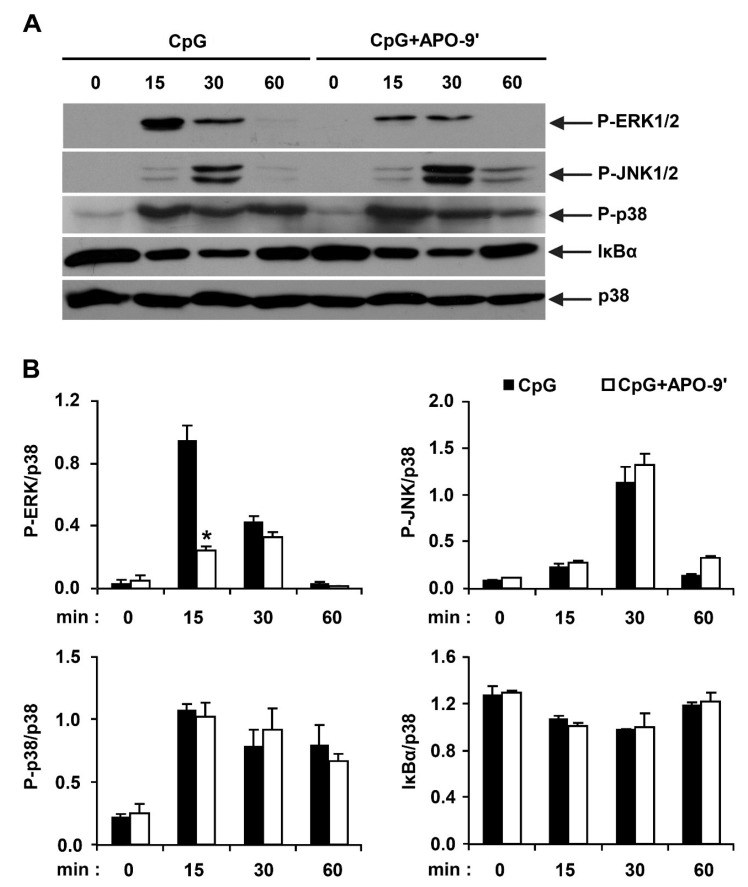
Effects of APO-9′ on the phosphorylation of MAPK and the degradation of IκBα by CpG DNA-stimulated BMDMs. (**A**) Cells were pre-treated with or without APO-9′ (20 µM) for 1 h before stimulation with CpG DNA (1 µM). Total cell lysate was obtained at the indicated time intervals. Western blot analysis was performed on the cell lysate to assess phosphorylation of ERK, JNK and p38 and the degradation of IκBα. Total p38 MAPK was taken as the loading control. Data are representative of three independent experiments. (**B**) Phosphorylation of ERK, JNK and p38 and IκBα protein expression was quantified using scanning densitometry, and the band intensities were normalized by that of total p38 protein. APO-9′, Apo-9′-fucoxanthinone. * *p* < 0.05 *vs.* APO-9′-untreated cells in the presence of CpG DNA.

Stimulation of TLR9 by CpG DNA triggers the activation of NF-κB and MAPK pathways, leading to the production of pro-inflammatory cytokines [1,27]. Hence, by Western blot analysis, we investigated the effects on MAPK phosphorylation and NF-κB activation in CpG DNA-stimulated BMDMs, with and without APO-9′ treatment (Figure 5). All three MAPK became phosphorylated in BMDMs stimulated with CpG DNA. ERK1/2, JNK1/2 and p38 phosphorylation was detected between 15 to 30 min of CpG DNA-stimulation. ERK1/2 and JNK1/2 phosphorylation returned to baseline levels within 60 min of CpG DNA-stimulation. APO-9′ pre-treatment in the presence of CpG DNA showed strong inhibition of ERK1/2 phosphorylation (Figure 5A,B). Stimulation of TLR leads to phosphorylation of IκB by IκB kinase, and the subsequent ubiquitination and degradation of IκB results in activation of NF-κB [1]. Activation of NF-κB was assessed indirectly by the degradation of IκBα. CpG DNA-stimulation induced IκBα degradation within 30 min of stimulation (Figure 5A,B). The amount of IκBα protein returned to baseline levels after 60 min post-stimulation. However, APO-9′ pre-treatment did not block IκBα degradation in CpG DNA-stimulated BMDMs (Figure 5A,B). Taken together, these data suggest that APO-9′ can inhibit CpG-stimulated ERK1/2 phosphorylation in BMDMs.

### 2.4. APO-9′ Treatment Inhibited AP-1 Reporter Activity in HEK293T Cells

**Figure 6:**
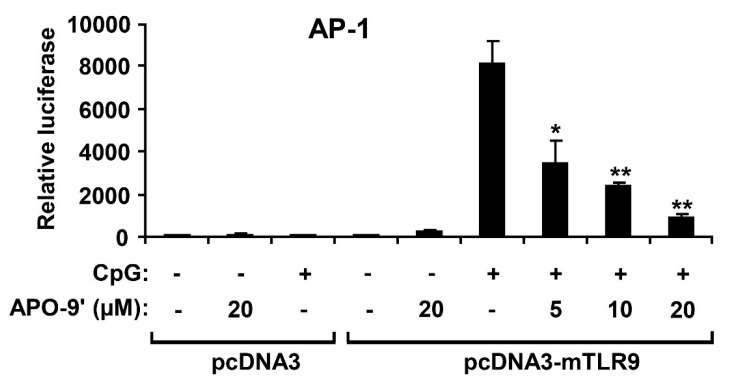
Effects of APO-9′ on AP-1 reporter activity in HEK293T cells. HEK293T cells were transfected with a murine with an empty vector (pcDNA3) or a TLR9-expressing plasmid (pcDNA3-mTLR9) and then treated with APO-9′ for 1 h before stimulation with CpG DNA (1 μM). Cell lysates were prepared, luciferase activity was assayed by the dual luciferase reporter assay and the results were expressed as relative luciferase. Data are representative of three independent experiments. APO-9′, Apo-9′-fucoxanthinone. * p < 0.05, ** p < 0.01 *vs.* APO-9′-untreated cells in the presence of CpG DNA.

Activation of MAPK induces increased AP-1 transcriptional activity, which, in turn, leads to expression of multiple AP-1-associated genes, including pro-inflammatory cytokines [27,28]. To investigate whether the APO-9′ had an inhibitory effect on CpG DNA-stimulated AP-1 transcriptional activity, an AP-1 reporter gene assay was conducted (Figure 6). The HEK293T cells transfected with empty pcDNA3 showed little AP-1-dependent luciferase activity on CpG DNA-stimulation. In contrast, the HEK293T cells transfected with pcDNA3-mTLR9 exhibited robust AP-1-dependent luciferase activity on CpG DNA-stimulation. However, APO-9′ pre-treatment showed strong dose-dependent inhibition of AP-1-dependent luciferase activity in HEK293T cells transfected with pcDNA3-mTLR9 (Figure 6). Therefore, this data suggest that APO-9′ has an inhibitory effect on TLR9-dependent AP-1 activation on CpG DNA-stimulation.

### 2.5.APO-9′ Treatment Did Not Inhibit NF-κB Reporter Activity in HEK293T Cells

**Figure 7:**
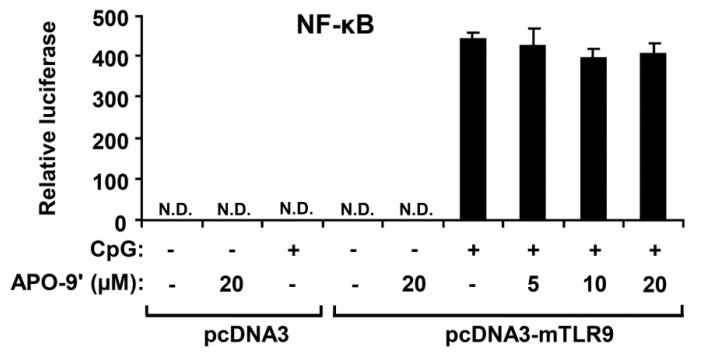
Effects of APO-9′ on NF-κB reporter activity in HECK293T cells. HEK293T cells were transfected with a murine with an empty vector (pcDNA3) or a TLR9-expressing plasmid (pcDNA3-mTLR9) and then treated with APO-9′ for 1 h before stimulation with CpG DNA (1 μM). Cell lysates were prepared, luciferase activity was assayed by the dual luciferase reporter assay and the results were expressed as relative luciferase. Data are representative of three independent experiments. APO-9′, Apo-9′-fucoxanthinone; N.D., not detectable.

Activation of the NF-κB pathway results in nuclear translocation and binding of this transcriptional factor to its target promoter sites [28]. To examine whether the APO-9′ has an inhibitory effect on CpG DNA-stimulated NF-κB transcriptional activity, an NF-κB reporter gene assay was preformed (Figure 7). The HEK293T cells transfected with empty pcDNA3 showed no NF-κB-dependent luciferase activity on CpG DNA-stimulation. The HEK293T cells transfected with pcDNA3-mTLR9 exhibited robust NF-κB-dependent luciferase activity on CpG DNA-stimulation. In accordance with Western blot analysis, APO-9′ pre-treatment did not show inhibition of NF-κB-dependent luciferase activity in HEK293T cells transfected with pcDNA3-mTLR9 (Figure 7). Hence, this data suggest that APO-9′ has no inhibitory effect on TLR9-dependent NF-κB activation on CpG DNA-stimulation.

### 2.6. Effect of APO-9′ on the Production of iNOS in CpG DNA-Stimulated RAW264.7 Cells

**Figure 8:**
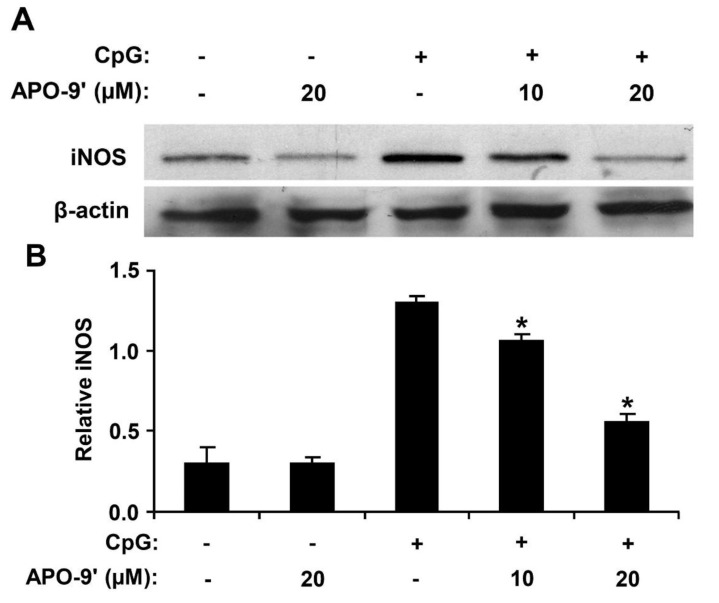
Effect of APO-9′ on the production of inducible nitric oxide synthase (iNOS) in CpG DNA-stimulated RAW264.7 cells. (**A**) RAW264.7 cells were pre-treated or not treated with APO-9′ at the indicated doses for 1 h before stimulation with CpG DNA (1 μM). The protein levels of iNOS were determined by Western blot analysis. β-actin was taken as the loading control. (**B**) iNOS protein expression was quantified using scanning densitometry and normalized by control protein. APO-9′, Apo-9′-fucoxanthinone. * p < 0.05 *vs.* APO-9′-untreated cells in the presence of CpG DNA.

To investigate the effect of APO-9′ on the production of iNOS in CpG DNA-stimulated RAW264.7 cells, Western blot analysis was used. Cell viability was simultaneously determined by using colorimetric MTT assay, and as a result, APO-9′ had no effect on the viability of RAW264.7 cells at the indicated concentrations (data not shown). CpG DNA-stimulation showed a robust increase in the production of iNOS in RAW264.7 cells (Figure 8). However, APO-9′ pre-treatment substantially inhibited iNOS expression in CpG DNA-stimulated RAW264.7 cells (Figure 8). Hence, this data suggests that APO-9′ has an inhibitory effect on the production of iNOS.

## 3. Discussion

In the present study, the anti-inflammatory effects of SME and APO-9′, a principal ingredient of SME, were investigated. To the best of our knowledge, this is the first study to demonstrate that APO-9′ isolated from *S. muticum* has the potential for medicinal application in inflammatory diseases.

Pro-inflammatory cytokines, such as IL-12 p40, IL-6 and TNF-α by activated BMDMs and BMDCs play a critical role in host defense and inflammatory response [1]. IL-12 p40 performs important immunoregulatory activities and is a key cytokine in Th1-mediated autoimmune responses, so downregulation of IL-12 p40 production by SME and APO-9′ may be helpful in combating IL-12 p40-associated autoimmune diseases [29,30]. IL-6 has broad cellular and physiological responses, including hematopoiesis, inflammation, regulation of cell growth, proliferation and differentiation [31,32]. However, IL-6 has also been linked to different diseases, such as diabetes, atherosclerosis, depression, Alzheimer's disease and rheumatoid arthritis [33]. In the present study, pre-treatment of SME and APO-9′ showed a strong dose-dependent inhibition of IL-6 production in CpG DNA-stimulated BMDMs and BMDCs. Therefore, downregulation of IL-6 production by SME and APO-9′ may have potentials in anti-inflammatory and anti-cancer application.

TNF-α is primarily produced by monocytes and macrophages and has critical immunoregulatory roles that are required to maintain immune homeostasis. However, overproduction of TNF-α has been associated with autoimmune disorders, such as rheumatoid arthritis and Crohn's disease [34,35]. In the present study, APO-9′ exhibited a significant inhibitory effect on the production of TNF-α in CpG DNA-stimulated BMDMs and BMDCs, suggesting that it may have the potential to ameliorate TNF-α-associated diseases. Hence, the strong inhibitory properties of SME and APO-9′ against IL-12 p40 and IL-6 production warrant further studies concerning potentials in future anti-inflammatory application.

Stimulation of TLR9 by CpG DNA triggers the activation of both MAPKs and NF-κB pathways, leading to production of inflammatory cytokines [36,37]. In the present study, pre-treatment of APO-9′ showed no inhibition of NF-κB activation as determined by IκBα degradation. Thus, our result suggests that the anti-inflammatory activity of APO-9′ may be independent of NF-κB activation. Its pre-treatment resulted in strong inhibition of the phosphorylation of ERK1/2 and AP-1 reporter activity. Taken together, these findings suggest that inhibition of CpG DNA-stimulated pro-inflammatory cytokines production by APO-9′ may correlate with blockage of the AP-1-dependent pathway.

It was reported that fucoxanthin isolated from brown algae exhibits an anti-inflammatory effect on the production of TNF-α, IL-6 and iNOS in lipopolysaccharide-stimulated RAW264.7 macrophages [21]. Similarly, we found that APO-9′ exhibited an inhibitory effect on CpG-induced pro-inflammatory cytokine production and iNOS expression.

Overexpression of iNOS is associated with many serious diseases, such as septic shock, arthritis, chronic inflammatory diseases and autoimmune diseases [13,38,39]. In the present study, pre-treatment of APO-9′ showed a significant inhibitory effect on CpG-induced iNOS expression. Taken together, APO-9′-mediated anti-inflammatory activity represents a potential therapeutic use of the compound for inflammatory diseases.

## 4. Experimental Section

### 4.1. Preparation of *S. muticum* Extract (SME)

Thalli of *S. muticum* were collected on Jeju Island, Korea. A voucher specimen has been deposited at the herbarium of Jeju Biodiversity Research Institute. The materials for extraction were cleaned, dried at room temperature for 1 week and ground into a fine powder. The dried alga (100 g) was extracted with 80% ethanol (EtOH; 2 L) at room temperature for 24 h and then evaporated under a vacuum. The evaporated ethanol extract (21 g) was suspended in water (4 L).

### 4.2. Isolation of Apo-9′-fucoxanthinone from SME

The shade-dried whole plant of brown algae *S. muticum* (2 kg) was extracted with 80% aqueous methanol under stirring for 2 days at room temperature. The filtrate was concentrated under reduced pressure and dried to give powder. The powdered extract was then suspended in water (1.0 L) and successively partitioned into n-hexane, methylene chloride, ethyl acetate, n-butanol and water fractions.

The methylene chloride fraction (5.4 g) was chromatographed over celite with *n*-hexane, methylene chloride, ethyl acetate and methanol successively (hexane/CH_2_Cl_2_ 1:0, 10:1, 5:1, 2:1, 0:1, CH_2_Cl_2_, EtOAc, MeOH). The (hexane/CH_2_Cl_2_ 1:0) fraction was chromatographed normal phase silica gel (3 cm × 70 cm) eluted with hexane/ethyl acetate/methanol (2:1:0.1). The fraction provided apo-9′-fucoxanthinone (APO-9′, 1.8 mg). The purified compound was identified by comparing its ^1^H- and ^13^C-NMR data with those reported in the literature [24,40]. APO-9′ was dissolved in DMSO (Amresco, Solon, OH, USA).

### 4.3. Mice

Six-week-old female C57BL/6 mice were purchased from Orient Bio Inc. (Seongnam, Korea) and maintained under specific pathogen-free conditions. All mice were maintained and used in accordance with institutional and National Institutes of Health guidelines. All animal procedures were approved by and performed according to the guidelines of the Institutional Animal Care and Use Committee of Jeju National University, Jeju, Korea (#2010-0028).

### 4.4. Cell Cultures and Measurement of Cytokine Production

To grow BMDMs and BMDCs, wild-type 6-week-old female C57BL/6J mice were used as previously described [41]. Briefly, bone marrow from tibia and femur was obtained by flushing with DMEM (Gibco, NY, USA), and bone marrow cells were cultured in RPMI 1640 (Gibco, NY, USA) medium containing granulocyte-macrophage colony-stimulating factor for dendritic cells generation. In the meantime, the bone marrow cells were cultured for macrophages in DMEM medium containing 20% heat-inactivated FBS, 30% L929 cell culture supernatant containing macrophage colony-stimulating factor and 1% penicillin-streptomycin (Gibco, NY, USA). For BMDCs, on day 6 of incubation, the cells were harvested and seeded in 48-well plates at a density of 1 × 10^5^ cells/0.5 mL and, then, treated with SME or APO-9′ for 1 h before stimulation with CpG DNA (1 μM). For BMDMs, on day 6 of incubation, the cells were harvested and seeded in 48-well plates at a density of 1 × 10^5^ cells/0.5 mL and, then, treated with the APO-9′ for 1 h before stimulation with CpG DNA (1 μM). Supernatants were harvested 18 h after stimulation. The concentrations of murine IL-12 p40, IL-6 and TNF-α in the culture supernatants were determined by enzyme-linked immunosorbent assay (ELISA) (BD PharMingen, San Jose, CA, USA, R&D system, MN, USA), according to the manufacturer’s instructions.

### 4.5. Cell Viability Assay

To assess cell viability, the standard procedure of 3-(4,5-dimethyl-2,5 thiazolyl)-2,5 diphenyl tetrazolium bromide (MTT) assay was used. Briefly, the cells at a concentration of 4 × 10^4^ cells were seeded on a 96-well culture plate. After incubation for 1 h at 37 °C, cells were treated with SME or APO-9′ at various concentrations for 18 h. Cells were added to 0.2 mg MTT (Sigma, St. Louis, MO, USA) and, then, incubated for 4 h at 37 °C. The plate was centrifuged, and the supernatants were aspirated. The formazan crystals in each well were dissolved in 250 μL dimethyl sulfoxide (DMSO) (Amresco, Solon, OH, USA). Absorbance was measured at a wavelength of 540 nm.

### 4.6. Western Blot Analysis

This was performed using standard techniques as previously described [42]. Briefly, BMDMs and RAW264.7 cells were dispensed to 60-mm culture dishes (Nunc, Roskilde, Denmark) at 4 × 10^6^ cells per dish and cultured for 24 h at 37 °C. The cells were pre-treated with or without APO-9′ (20 µM) for 1 h before treatment with CpG DNA at the indicated time points. The cells were collected and, then, lysed in lysis buffer (PRO-PREP lysis buffer, iNtRON Biotechnology, South Korea). A protein sample (30 µg) was subjected to electrophoresis in 10% SDS-polyacrylamide gels and transferred to a polyvinylidene fluoride membrane (Bio-Rad, Hercules, CA, USA). The membrane was incubated with 1/1,000-diluted rabbit polyclonal antibodies that specifically recognize phospho-p44/42 (P-ERK1/2), p44/42 MAPK, phospho-p38, p38 MAPK and phospho-SAPK/JNK, SAPK/JNK, IκBα (Cell Signaling Technology, Danvers, MA, USA), iNOS and β-actin (Santa Cruz Biotechnology, Santa Cruz, CA, USA). After washing, the membrane was incubated with a horseradish peroxidase-linked goat anti-rabbit IgG (Cell Signaling Technology), and immunoactive bands were detected as previously described [42].

### 4.7. Luciferase Assay

For AP-1 and NF-κB reporter assays, HEK293T cells were plated in 24-well plates and grown overnight as previously described [43]. AP-1 and NF-κB reporters and the pRLnull plasmid used in the luciferase assay were the kind gift of Dr. K. Kobayashi. Murine TLR9 expressing plasmid (pcDNA3-mTLR9) was similarly provided by Dr. R. Medzhitov. HEK293T cells were transfected with AP-1 or the NF-κB reporter gene together with pRLnull and pcDNA3-mTLR9 using Fugene 6 (Roche Diagnostics GmbH, Mannheim, Germany). Cells were then further incubated for 24 h and, then, pre-treated with APO-9′ for 1 h before stimulation with CpG DNA (1 μM). After further incubation for 18 h, cells were lysed in a passive lysis buffer (Promega, Madison, USA), and firefly luciferase *versus* Renilla activities were measured using a dual luciferase reporter assay system (Promega, Madison, WI, USA).

### 4.8. Data Analysis

All experiments were performed at least 3 times, and the data are presented as the mean ± the standard deviation (SD) of 3 independent experiments. One-way ANOVA was used for comparison between the treated and the control groups. *p* < 0.05 was considered statistically significant.

## 5. Conclusions

In conclusion, we found that SME and APO-9′ had an inhibitory effect on the production of pro-inflammatory cytokines by attenuating TLR9-dependent AP-1 activation. These results suggest that SME and APO-9′ might be beneficial in the treatment of inflammation- and autoimmune-associated diseases. Therefore, further studies are required on the detailed mode of action and *in vivo* efficacy of SME and APO-9′.

## Abbreviations

IC_50_: half maximal inhibitory concentration
N.D.: not detectable
NMR: nuclear magnetic resonance
AP-1: activator protein-1
DMSO: dimethyl sulfoxide
DMEM: Dulbecco’s modified Eagle’s medium
RPMI: Roswell park memorial institute medium
FBS: fetal bovine serum
SDS: sodium dodecyl sulfate
